# Effects of handrail hold and light touch on energetics, step parameters, and neuromuscular activity during walking after stroke

**DOI:** 10.1186/s12984-015-0051-3

**Published:** 2015-08-23

**Authors:** T. IJmker, C. J. Lamoth, H. Houdijk, M. Tolsma, L. H. V. van der Woude, A. Daffertshofer, P. J. Beek

**Affiliations:** MOVE Research Institute Amsterdam, Department of Human Movement Sciences, Faculty of Behavioural and Movement Sciences, VU University Amsterdam, van der Boechorststraat 9, 1081 BT Amsterdam, The Netherlands; Heliomare Rehabilitation, Research and Development, Relweg 51, 1949 EC Wijk aan Zee, The Netherlands; University of Groningen, University Medical Center Groningen, Center for Human Movement Sciences, Center for Rehabilitation, Antonius Deusinglaan 1, 9713AV Groningen, The Netherlands

**Keywords:** Energy cost, Stroke, Neuromuscular function, Gait, Balance support

## Abstract

**Background:**

Holding a handrail or using a cane may decrease the energy cost of walking in stroke survivors. However, the factors underlying this decrease have not yet been previously identified. The purpose of the current study was to fill this void by investigating the effect of physical support (through handrail hold) and/or somatosensory input (through light touch contact with a handrail) on energy cost and accompanying changes in both step parameters and neuromuscular activity. Elucidating these aspects may provide useful insights into gait recovery post stroke.

**Methods:**

Fifteen stroke survivors participated in this study. Participants walked on a treadmill under three conditions: no handrail contact, light touch of the handrail, and firm handrail hold. During the trials we recorded oxygen consumption, center of pressure profiles, and bilateral activation of eight lower limb muscles. Effects of the three conditions on energy cost, step parameters and neuromuscular activation were compared statistically using conventional ANOVAs with repeated measures. In order to examine to which extent energy cost and step parameters/muscle activity are associated, we further employed a partial least squares regression analysis.

**Results:**

Handrail hold resulted in a significant reduction in energy cost, whereas light touch contact did not. With handrail hold subjects took longer steps with smaller step width and improved step length symmetry, whereas light touch contact only resulted in a small but significant decrease in step width. The EMG analysis indicated a global drop in muscle activity, accompanied by an increased constancy in the timing of this activity, and a decreased co-activation with handrail hold, but not with light touch. The regression analysis revealed that increased stride time and length, improved step length symmetry, and decreased muscle activity were closely associated with the decreased energy cost during handrail hold.

**Conclusion:**

Handrail hold, but not light touch, altered step parameters and was accompanied by a global reduction in muscle activity, with improved timing constancy. This suggests that the use of a handrail allows for a more economic step pattern that requires less muscular activation without resulting in substantial neuromuscular re-organization. Handrail use may thus have beneficial effects on gait economy after stroke, which cannot be accomplished through enhanced somatosensory input alone.

**Electronic supplementary material:**

The online version of this article (doi:10.1186/s12984-015-0051-3) contains supplementary material, which is available to authorized users.

## Background

Regaining the ability to walk independently is an important goal in the rehabilitation of stroke survivors. Only 60 % of all stroke survivors eventually attain this goal to the level of community walking [1]. An important limiting factor in this regard is the substantial metabolic cost of hemiparetic gait, which can be more than two times larger than in healthy subjects [2–4], and which is predictive of community ambulation [5]. We have previously shown that an increased (metabolic) effort to control balance contributes to this decreased gait economy [6], and that this cost can be reduced considerably by providing balance support in the form of a handrail or cane [7].

Using a handrail or cane may have biomechanical and/or somatosensory advantages that could facilitate balance control. Biomechanically, the use of a handrail or cane increases the base of support, resulting in greater margins of stability, and enables one to generate corrective forces via the hands to compensate for perturbations [8]. Apart from this biomechanical advantage, the use of a handrail or cane may provide additional somatosensory (tactile and proprioceptive) information about body orientation and movement relative to the point of contact [8, 9]. This may reduce sensory noise/uncertainty and might therefore lead to better balance control [9, 10]. There is experimental support that, even in the absence of additional biomechanical support, the mere contact of fingertips or hand with a stable support surface can decrease the excursion of the center of mass during standing and walking [9–13]. This decrease matched that observed with firm handrail hold in healthy participants and stroke survivors. This suggests that enhanced somatosensory information may add to the mechanical stabilization through holding a handrail, which in turn may result in a decreased energy cost of walking after stroke.

To unravel the factors underlying the differential effects of handrail hold and light touch on the energy cost of walking, it is imperative to investigate which gait parameters alter in line with metabolic changes and which neuromuscular modifications might engender these effects. In stroke survivors, handrail or cane use yields increased stride length and time as well as decreased cadence and step width and variability [14, 15]. These changes may be linked to an improved gait efficiency through a more optimal step length/frequency combination [16, 17], and lower step-to-step transition costs with a smaller step width [18]. Using a handrail or cane may also improve gait symmetry [19, 15], which may also contribute to enhanced gait economy [20].

Effects of holding a handrail or cane have also been examined in terms of changes in neuromuscular control as reflected in altered amplitude and timing of muscle activation. Some studies reported decreases in EMG burst duration and a decrease in amplitude of several lower limb muscles during cane use [21, 22]. Furthermore, a decrease in the variability of EMG profiles of the lower leg muscles has been found as a result of handrail support, which indicates a more consistent timing of muscle activity possibly relating to increased (lateral) gait stability [23, 24]. Reduced EMG amplitude and more accurate timing of muscle activity may reflect improved economy [25]. In contrast, other studies reported no effect of firm handrail hold or light touch contact with a cane on muscle activity [26, 27], while light touch contact has even been shown to result in higher activation amplitudes than force contact [12, 26].

As of yet, it is unclear whether somatosensory and/or biomechanical aspects of handrail or cane use affect the energy cost of walking after stroke, nor whether altered step parameters and/or altered neuromuscular control are responsible for this effect. Our research aims were therefore twofold: 1) to compare the effects of light touch contact with a handrail and firm handrail hold on the energy cost of walking, step parameters, and muscle activity (in terms of amplitude and timing) in stroke survivors, and 2) to examine which changes in step parameters and muscle activity are associated with the observed changes in energy cost. To evaluate changes in muscle activation amplitude and timing we used a principal component analysis (PCA), since this method allows for studying patterns of multivariate muscle activation instead of looking at isolated muscle activities alone.

## Methods

### Participants

Fifteen stroke survivors from the in- and outpatient stroke unit of rehabilitation center Heliomare, Wijk aan Zee, The Netherlands, agreed to participate in the study. All participants received therapy for stroke-related gait impairments at the time of the experiment, were able to walk independently on a treadmill for at least 5 minutes, and scored between 3–5 on the Functional Ambulatory Category (FAC). People with cognitive, communicative, or non-stroke related orthopedic or neurologic impairments, or a contraindication for moderate exercise, were excluded from the study. Descriptive characteristics of the study population are presented in Table 1. All participants received written and verbal information about the experiment and provided a written informed consent. The Medical Ethical Committee of the VU University Medical Center, Amsterdam, The Netherlands, approved the experiment prior to conduction.

**Table 1:** Descriptive characteristics of study population (*N* = 15)

Characteristics	Values
Gender (male/female)	12/3
Age (yrs)	57.5 ± 10.16
Weight (kg)	82.0 ± 18.45
BMI^a^	25.5 ± 5.31
Etiology (infarct/hemorhage)	13/2
Lesion side (left/right)	5/10
Time since stroke (days)	69.5 ± 38.39
AFO^b^ (yes/no)	5/10
Walking aid (none/cane/walker/quadcane)	3/7/4/1
Preferred walking speed on the treadmill (m · s^−1^)	0.52 ± .19
ABC-score^c^	67.7 ± 19.69
FAC^d^ (3/4/5)	1/3/11
BBS^e^	50.0 ± 5.93

^a^ = body mass index; ^b^ = ankle foot orthosis; ^c^ = activities specific balance confidence score; ^d^ = functional ambulatory category; ^e^ = Berg balance score. Values are mean ± SD unless otherwise indicated

### Study protocol

Participants visited the lab twice. The first session was used to familiarize them with walking on a treadmill under the aforementioned experimental conditions, while the actual measurements were conducted in the second session. At the end of the first session, the participant’s preferred walking speed while walking without handrail use was established following a previously employed protocol [7]. This speed was subsequently used in the experimental trials. The protocol consisted of three experimental trials: walking with handrail hold (HOLD), walking with light touch handrail contact (TOUCH), and walking without handrail contact (NORM). The randomly offered experimental trials lasted 5 minutes each in order to ensure steady state oxygen consumption during the second half of the trial. Since some participants were unable to walk for 5 minutes on the treadmill, trial duration was reduced to 4 minutes for those participants. In the TOUCH condition, participants were instructed to lightly touch an aluminum plate mounted on the handrail with the fingertips of their non-paretic hand without exceeding a force of 5 N in the vertical (V), anteroposterior (AP), and mediolateral (ML) direction. The amount of force exerted in each direction was monitored during the trial and, if necessary, verbal feedback was provided to reduce the amount of force. In the HOLD condition participants were instructed to hold the handrail at all times without any specific instruction as to how to use the handrail. The paretic arm was allowed to hang freely during the trial, unless participants preferred to carry their arm in a sling. Participants wore a harness during all trials for safety, which did not provide any body weight support.

### Equipment

Participants walked on an instrumented treadmill with an embedded force plate (C-Mill, ForceLink, Culemborg, The Netherlands; size 1 m × 1.5 m, sampling rate 100 Hz), from which step parameters were derived off-line. Oxygen consumption (*VO*_2_) and respiratory exchange ratio (*RER*) were measured breath-by-breath using open circuit respirometry (Oxycon delta, CareFusion, San Diego, USA). An instrumented handrail, equipped with two 6-DOF force sensors (AMTI, Watertown, USA, sampling rate 100 Hz), was placed on the non-paretic side of the participant to measure the forces exerted on the rail. Muscle activity of the following 8 muscles of both legs were recorded using surface electromyography and sampled at a rate of 1 kHz (TMSi, Enschede, The Netherlands); *m*. gastrocnemius medialis, *m*. tibialis anterior, *m*. peroneus longus, *m*. rectus femoris, *m*. vastus lateralis, *m*. semitendinosus, *m*. tensor fascia latae, *m*. gluteus medius. EMG recordings were made with disposable Ag/AgCl electrodes (∅ 20 mm) with an inter-electrode distance of 10 mm after standard skin preparations following SENIAM recommendations [28].

### Data analysis

#### Energy cost

After visually inspecting the data to ensure steady state oxygen consumption, gross energy expenditure (*EE*_gross_) was determined during the final 90s each trial via the *RER* and the oxygen consumption ${\overset{.}{VO}}_2$ according to $E{E}_{\mathrm{gross}} = \left(4.960\kern0.5em RER+16.040\right)\kern0.5em {\overset{.}{VO}}_2$; see [29]. Energy expenditure at rest was subtracted from *EE*_gross_ to obtain net energy expenditure (*EE*_net_). Subsequently, *EE*_net_ was divided by body mass and walking speed to obtain net energy cost.

#### Spatiotemporal gait parameters

Data from the force plate of the treadmill were converted to center of pressure profiles (*COP*_AP_ and *COP*_ML_ in anteroposterior and mediolateral direction, respectively). The characteristic butterfly pattern of these profiles (*COP*_AP_ over *COP*_ML_ plots) served to identify initial contact and toe-off through peak detection [30]. Instances of initial contact were used to determine mean and variability of stride time, length, and width. Step time was defined as the time between two consecutive initial contacts. Stride time was defined as the sum of the corresponding left and right steps. Stride/step length were derived by multiplying belt speed by stride/step time and correcting for the difference in *COP*_AP_ between the two initial contacts. Step width was defined as the absolute distance in *COP*_ML_ at two consecutive initial contacts. Temporal and spatial symmetry were set as 2 ⋅ *T*_*np*_/(*T*_*np*_ + *T*_*p*_) or 2 ⋅ *L*_*np*_/(*L*_*np*_ + *L*_*p*_) where *T*_*np*_/*L*_*np*_ and *T*_*p*_/*L*_*p*_ are the step time/length of the non-paretic and paretic leg, respectively. A value of 1 indicates perfect symmetry, while a value > 1 indicates a higher value for the non-paretic leg and a value < 1 indicates a higher value for the paretic leg.

#### Muscle activity

Differences in EMG activity patterns between conditions were analyzed in terms of amplitude and timing constancy of the muscle coordination pattern, and muscle co-activation to assess both quantitative and qualitative changes in muscle activation. To detect differences in the amplitude and constancy of the coordination pattern instead of in isolated muscles, we first reduced the data to major co-varying modes or principal components, using principal component analysis (PCA) [31, 32].

We selected the EMG of all complete strides from the last 90 seconds per trial, starting from foot contact of the non-paretic leg. These signals were first high-pass filtered (2^nd^ order, bi-directional Butterworth, cut-off frequency 20 Hz), then full-wave rectified via the absolute value of the corresponding analytic signal constructed via the Hilbert transform, and finally low-pass filtered (2^nd^ order, bi-directional Butterworth, cut-off frequency 5 Hz) to estimate the linear envelope. For the PCA, signals were mean-centered (DC-removal). We concatenated the signals of all conditions (*c* = 1 … 3) and participants (*p* = 1 … 15), generating a dataset *X*_*m*,*k*_ consisting of 16 time series (*m* = muscle 1 to 16) of *N* = ∑_*p* = 1_^15^ ∑_*c* = 1_^3^ *N*^(*p*,*c*)^ = 4, 145, 597 samples each. Note that one can identify individual signals in that dataset *X*_*m*,*k*_ by using *k* ∈ *T*^(*p*,*c*)^ as the sample subset (or time interval) when referring to participant *p*’s muscle *m* in condition *c*.

Next, we estimated the covariance matrix of *X* [33], normalized by its trace, and computed the corresponding eigenvectors *v*^(*j)*^ and eigenvalues λ_j_ that determine the principal component *j*. An element *n* of eigenvector *v*^(*j*)^ = (*v*_1_^(*j*)^, …, *v*_*n*_^(*j*)^ , …, *v*_16_^(*j*)^) represents the degree to which the EMG signal of muscle *m* = *n* contributed to the principal component *j*. An eigenvalue (λ_j_) represents the amount of variance of the original data explained by principal component *j*. The eigenvectors and eigenvalues were sorted in descending order of eigenvalue. The number of to-be-considered modes *J* was determined by visual inspection of the eigenvalue spectrum, using a discontinuous decrease in eigenvalue on a log-log scale as cut-off criterion. The time course *Y*_*k*_^(*j*)^ along mode *j* was defined by projecting the original data set onto *v*^*(j)*^, i.e. by using *Y*_*k*_^(*j*)^ = *Σ*_*m* = 1_^16^*v*_*m*_^(*j*)^*X*_*m*,*k*_.

From the projections *Y*_*k*_^(*j*)^ we distilled two outcomes: (*i*) the degree to which a mode contributed to the overall EMG pattern of a certain participant and condition by estimation of the mean amplitude of a mode using the root-mean squared value (RMS); and (*ii*) the constancy of the muscle coordination in terms of proper timing, quantified by the variance of the relative phase between modes (*V*) [34]. For the calculation of RMS we applied PCA after normalizing the EMGs per subject to the standard deviation determined during the corresponding NORM condition, which hence served as ‘reference trial’. The root-mean-squared value was calculated for each participant *p* and condition *c* via $RM{S}_{p,c}^{(j)}\kern0.5em =\kern0.5em \sqrt{\frac{1}{N^{\left(p,c\right)}}{\varSigma}_{k\in {T}^{\left(p,c\right)}}\kern0.5em {\left({Y}_k^{(j)}\right)}^2}$.

A drop of *RMS*_*p*,*c*_^(*j*)^ over conditions in a specific mode *j* would indicate a decrease in the contribution of mode *j* to the overall EMG pattern and therefore a qualitative change in muscle activation. In contrast, a decrease in *RMS*_*p*,*c*_^(*j*)^ of all modes *j* = 1, …, *J*, would reflect a global decrease in muscle activation.

To assess the constancy of muscle activation timing we determined the variance of the relative phase. As our focus was on the timing we reduced amplitude effects by dividing the data of every condition by its own standard deviation (i.e., *z*-scoring the data). We also time normalized the data to 100 samples per stride to remove temporal differences, and filtered them to a narrow frequency band around the stride frequency (2^nd^-order, bi-directional Butterworth, 0.25-1.75 times stride frequency); by this we reduced possible confounding effects of higher harmonics when estimating the phase. For every mode *j* = 1, …, *J* the instantaneous phase *φ*_*k*_^(*j*)^ was determined as the angle of the corresponding analytic signal constructed via Hilbert transform (also referred to as Hilbert phase). Circular normality is a prerequisite for reliable phase estimation. Therefore, we tested all possible pairs of relative phases for circular normality using Kuiper’s test against the von Mises distribution. This indicated that circular normality was only met for the relative phase between mode 1 and 2 (i.e. *Δφ*_*k*_ = *φ*_*k*_^(1)^ − *φ*_*k*_^(2)^). For this pair, circular variance [35] was estimated as ${V}_{p,c}\kern0.5em =\kern0.5em 1\kern0.5em -\kern0.5em \sqrt{{\left(\frac{1}{N^{\left(p,c\right)}}{\sum}_{k\in {T}^{\left(p,c\right)}}\kern0.5em  \sin \varDelta \varphi k\right)}^2+\kern0.5em {\left(\frac{1}{N^{\left(p,c\right)}}{\sum}_{k\in {T}^{\left(p,c\right)}}\kern0.5em  \cos \varDelta \varphi k\right)}^2}$.

The variance of the relative phase between two modes (0 ≤ *V* ≤ 1) provides an index of the constancy of the coordination pattern, with lower values indicating a more constant activation timing profile.

To determine whether changes in muscle activation amplitude could originate from altered co-activation, a co-activation index (CAI) was calculated on the original full-wave rectified and filtered EMG profiles. The CAI (in %) was calculated as the common area of activity of two antagonistic muscles [36].

$$ CA{I}_{m_1,{m}_2}^{\left(p,c\right)}\kern0.5em =\kern0.5em \frac{\sum_{k=1}^{\left(p,c\right)} \min \left({x}_{m_1,k}^{\left(p,c\right)},{x}_{m_2,k}^{\left(p,c\right)}\right)}{\frac{1}{2}{\sum}_{k=1}^{N^{\left(p,c\right)}}\left({x}_{m_1,k}^{\left(p,c\right)}+{x}_{m_2,k}^{\left(p,c\right)}\right)}\cdot 100\% $$

We used the (*m*_1_,*m*_2_) muscle pairs *m*. gastrocnemius medialis – *m*. tibialis anterior (GM_TA), *m*. tibialis anterior – *m*. peroneus longus (TA_PL), and *m*. rectus femoris – *m*. semitendinosus (RF_ST) of the paretic and non-paretic leg for each participant *p* and condition *c*. In two subjects *m*. gastrocnemius medialis of the paretic leg could not be accessed due to their orthosis, precluding computation of CAI for GM_TA for these participants.

### Statistical analysis

A repeated analysis of variance with condition [NORM, TOUCH, HOLD] as within-subjects factor was used to analyze the effect of condition on energy cost, step parameters (mean and variability of stride time, stride length and step width, and temporal and spatial symmetry), and muscle activity parameters (*RMS*, *V*, and *CAI*). Planned contrasts with Bonferroni correction for multiple comparisons were used to follow up on significant main effects of condition. The level of significance for all statistical analyses was set to *α* = .05.

To evaluate which gait changes were associated with the change in energy cost we performed a multivariate partial least squares regression (PLS). PLS uses principal component analysis followed by a regression step, and is particularly useful when the number of variables is large compared to the number of observations, as well as in the case of multi-collinearity, in which simple linear regression is not feasible (for a tutorial see [37, 38]). Briefly, the analysis identifies underlying latent factors (principal components), which best model the change in energy cost, thereby explaining as much of the covariance between the change in gait parameters and the change in energy cost as possible. Only the conditions (HOLD and/or TOUCH) that showed a statistically significant effect on energy cost were entered into the analysis. Before entering the analysis, difference scores between the condition in question and NORM were computed for energy cost and gait variables showing a significant effect of condition, and rescaled to unit variance. The quality of the model was assessed by the amount of variance explained by the model (*R*^*2*^). To quantify the relationship between the change in energy cost and the change in gait parameters, the regression coefficients and the variable *'importance on projection score'* (VIP) were evaluated. Variables with a VIP-score > 1.0 can be considered important for the model [37].

## Results

All subjects completed the protocol. Two participants had difficulty adhering to the forces allowed during TOUCH. However, since the overshoot was only minor, we decided to include these participants in the analysis. Further results pertaining to the forces exerted on the handrail can be found in the additional materials (Additional file 1: Figure S1 and Additional file 2: Table S1). Statistical results from the repeated measures ANOVA are presented in Table 2.

**Table 2:** Statistical results of repeated measures ANOVA

	Main effects of condition				TOUCH vs NORM			HOLD vs NORM		
	df	F	p	*η*_*p*_^2^ ^a^	F	p^b^	*η*_*p*_^2^ ^a^	F	p^b^	*η*_*p*_^2^ ^a^
Energy cost	2.00	8.35	.001	.323	.09	1.000	.006	8.46	.023*	.377
Spatiotemporal step parameters										
Stride time	1.09	17.38	.001*	.581	.54	.953	.037	19.54	.001*	.583
Stride time SD	1.03	0.91	.358	.075	.92	.708	.062	0.93	.705	.062
Stride time asymmetry	1.12	3.54	.075	.175	1.47	.490	.095	3.81	.142	.214
Stride length	1.17	35.13	.000*	.723	.82	.761	.055	40.53	.000*	.743
Stride length SD	1.04	1.46	.248	.100	1.36	.526	.089	1.59	.457	.102
Stride length asymmetry	1.42	7.32	.008*	.359	3.57	.160	.203	9.69	.015*	.409
Step width	2.00	32.18	.000*	.652	7.13	.037*	.337	52.17	.000*	.788
Step width SD	2.00	18.89	.000*	.551	.74	.805	.051	23.96	.000*	.631
Muscle activation parameters										
RMS mode 1	2.00	30.44	.000*	.685	1.37	.523	.089	68.87	.000*	.831
RMS mode 2	2.00	18.77	.000*	.573	1.62	.447	.104	18.44	.001*	.568
RMS mode 3	1.41	34.71	.000*	.713	1.65	.440	.105	42.97	.000*	.754
Relative phase variance^c^	2	6.41	.005*	.288	.84	0.75	.056	5.88	.059	.296
CAI GM_TA paretic	1.17	7.96	.011*	.399	.00	1.000	.000	8.93	.023*	.427
CAI TA_PL paretic	1.14	.54	.497	.043	.10	1.000	.008	.57	.927	.046
CAI RF_ST paretic	2	1.03	.373	.079	2.06	.354	.146	.11	1.000	.009
CAI GM_TA nonparetic	2	51.95	.000*	.812	.69	.846	.054	64.88	.000*	.844
CAI TA_PL nonparetic	2	6.46	.006*	.350	.41	1.000	.033	8.58	.025*	.417
CAI RF_ST nonparetic	2	1.82	.183	.132	2.02	.361	.144	2.48	.282	.171

^a^ = estimate of effect size, partial eta squared; ^b^ *p*-value corrected for multiple comparisons using Bonferoni correction. ^c^ = calculated using EMG normalized to unit variance over conditions, instead of normalized to NORM trial as reference

### Energy cost

**Fig. 1:**
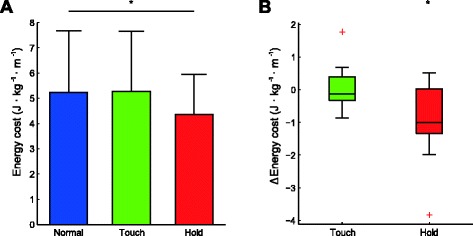
**a**. Effect of light touch and handrail hold on the net energy cost of walking. **b**. boxplot of the difference between Touch and Normal, and Hold and Normal. The central mark is the median, the edges of the box are the 25th (q^1^) and 75th (q^3^) percentiles. Whiskers extend to the last datapoint > *q* ^1^ − 1.5(*q* ^3^ − *q* ^1^) or < *q* ^3^ + 1.5(*q* ^3^ − *q* ^1^). Datapoints outside this range are shown as a red cross. * = significantly different from NORM at *p* < .05

HOLD caused a significant decrease in net energy cost (*p* = .023) of 11.8 % (0.86 J · kg^-1^ · m^-1^) on average compared to NORM, while TOUCH had no significant effect on energy cost (Fig. 1).

### Step parameters

**Fig. 2:**
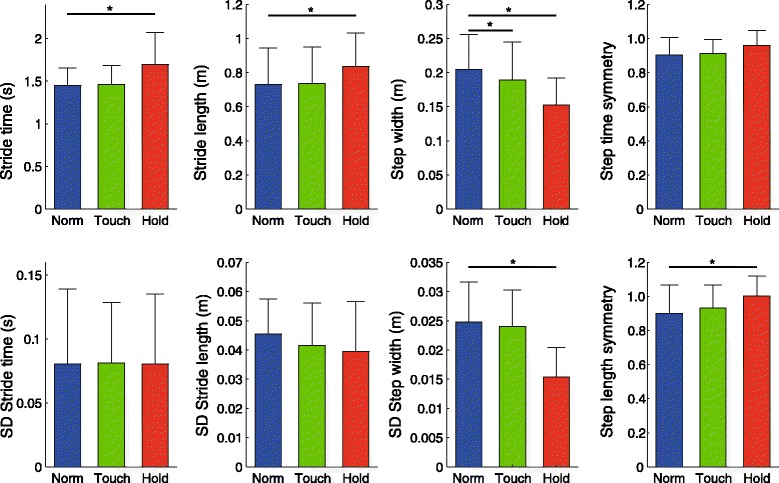
Effects of light touch and handrail hold on spatiotemporal step parameters. * = *p* < .05

As shown in Fig. 2, HOLD resulted in significant increases in stride time (16.1 %; *p* = .001) and length (16.3 %; *p* < .001), a significant decrease in step width (24.4 %; *p* < .001) and step width variability (35.5 %; *p* < .001), and improved step length symmetry (15.0 %; *p* = .015; Fig. 2). No significant effects were found for variability of stride time and stride length. TOUCH resulted in a significant decrease in step width of 7.7 % (*p* = .037; Fig. 2). No significant changes as a result of TOUCH were found for the other spatiotemporal parameters.

### Muscle activity

**Fig. 3:**
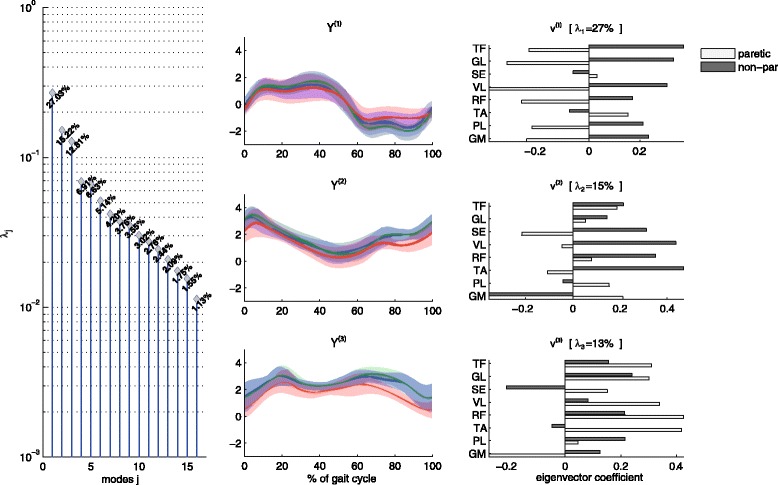
Eigenvalue spectrum λ_j_ (left panel), projections *Y*_*k*_^(*j*)^ (central panels) and eigenvectors *v* ^(*j*)^ = (*v*_1_^(*j*)^, …, *v*_*n*_^(*j*)^, …, *v*_16_^(*j*)^) (right panels) for the first three modes (*j*). Gait cycle for the central panel starts and ends with initial contact of the nonparetic leg

**Fig. 4:**
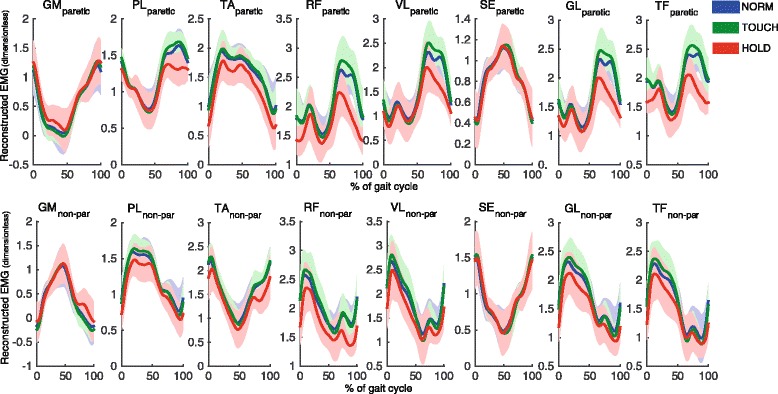
Reconstructed time normalized EMG patterns (dimensionless) of paretic (upper row) and non-paretic (lower row) leg, based on the three modes for each condition, averaged over strides and participants. Using the time courses *Y*_*k*_^(*j*)^ we reconstructed signals as superposition ${X}_{m,k}\approx {\tilde{X}}_{m,k}={\sum}_{j=1}^J{v}_m^{(j)}{Y}_k^{(j)}$. We further added the DC-values of the original EMGs to these time courses to generate EMG-like patterns. Solid lines indicate averages, shaded areas indicate SD over participants. Stride cycle starts and ends with initial contact of the nonparetic leg. GM = *m*. Gastrocnemius medialis; TA = *m*. Tibialis anterior; PL = *m*. Peroneus longus; RF = *m*. Rectus femoris; VL = *m*. Vastus lateralis; ST = *m*. Semitendinosus GL = *m.* Gluteus medius; TF = *m*. Tensor fascia latae

For both normalization methods, the PCA on the EMG data resulted in *J* = 3 relevant modes (Fig. 3). The eigenvector coefficients were similar for the two normalization methods. We therefore only sketch the overall outcome for normalization to the NORM trial. Together the three modes represented only 55 % of the variance in the original EMG dataset. Nonetheless, reconstructed EMG signals based on these three modes resembled EMG patterns during walking (Fig. 4) rather well. The first one (*v*^(1)^) contained activity of all muscles of the paretic and non-paretic leg, as revealed by the individual eigenvector coefficients (*v*_1_^(1)^, … *v*_*n*_^(1)^, …, *v*_16_^(1)^) shown in the upper right panel of Fig. 3. The corresponding time course *Y*_*k*_^(1)^ oscillated at the stride frequency (Fig. 3, upper central panel). The opposite signs of the eigenvector values of the muscles of the paretic and the non-paretic legs represented the alternating (opposite) activation patterns of left and right leg (i.e. a phase shift around 180 degrees). The second mode was mainly evident in the activation of the non-paretic muscles, with activity mostly present during the beginning of the stance phase and a small burst during swing (Fig. 3, middle central and right panels). The third mode was predominantly represented in the paretic muscles, with a biphasic pattern with a burst during paretic leg swing, and a prolonged activity during the stance phase of the paretic leg (Fig. 3, lower central and right panel). In Fig. 4 we also show the quality of the PCA data reduction by reconstructing EMG-like patterns based on the small set of relevant modes (*Y*_*k*_^(*j*)^ and *v*^(*j*)^with *j* = 1, …, 3).

**Fig. 5:**
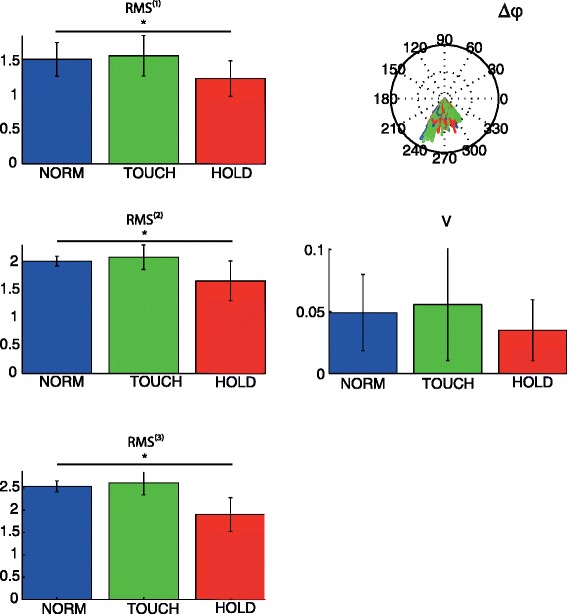
Changes in muscle activation patterns based on PCA analysis. Left column: root mean squared value (*RMS* ^(*j*)^) for first three modes for each condition averaged over subjects. Right column: Relative phase distribution plot (∆φ), for relative phase between mode 1 and 2, and variance (*V*) in relative phase between mode 1 and 2. As can be seen, the relative phase between mode 1 and 2 is centered between 240 and 300 (or: −60 and −100) degrees

The *RMS* of the projections of all three relevant modes showed a significant effect of condition. Planned contrasts revealed that for all three modes values were significantly lower during HOLD than during NORM (all *p*-values < .001), indicating a drop in amplitude in this condition (Fig. 5). This global amplitude drop is also visible in the reconstructed EMG patterns (cf. Fig. 4). In contrast, differences between TOUCH and NORM were not significant.

There was also a significant main effect of variance *V* of relative phase between mode 1 and 2 (*p* = .005; Fig. 5). Planned contrasts showed that the decrease in the variance of the relative phase between HOLD and NORM was nearly significant (*p* = .059), indicating an increased constancy in the timing of the coordination pattern during the HOLD condition. The difference between TOUCH and NORM was not significant (*p* = .752).

Significant main effects of condition on CAI of GM_TA of the paretic leg, and GM_TA and TA_PL of the non-paretic leg were found (all *p*-values < .05; Table 3). Planned contrasts showed that co-activation decreased for these muscle pairs in the HOLD condition compared to NORM. Again, the difference between TOUCH and NORM was not significant.

**Table 3:** Mean co-activation indices (SD)

	NORM	TOUCH	HOLD
GM_TA paretic	42.6 (13.96)	42.5 (12.51)	35.4 (15.84)
GM_TA nonparetic	41.1 (8.98)	40.7 (9.23)	32.1 (7.36)
TA_PL paretic	49.8 (17.19)	49.8 (16.37)	49.6 (16.42)
TA_PL nonparetic	50.1 (8.43)	49.5 (9.26)	46.1 (9.89)
RF_ST paretic	56.5 (7.93)	55.5 (8.79)	57.5 (6.28)
RF_ST nonparetic	57.0 (9.44)	55.7 (8.97)	54.5 (9.91)

GM_TA = *m*. Gastrocnemius medialis – *m*. Tibialis anterior; TA_PL = *m*. Tibialis anterior – *m*. Peroneus longus; RF_ST = *m*. Rectus femoris – *m*. Semitendinosus

### Association between changes in energy cost and changes in gait parameters

The regression analysis was only performed on the difference between HOLD and NORM because only HOLD resulted in significant changes in energy cost. The optimal model from the PLS regression contained a single latent factor which explained 70.5 % of the overall variance in the change in energy cost due to handrail hold. VIP scores and regression coefficients for the gait parameters can be found in Table 4. The most important step parameters associated with a decrease in energy cost (VIP score > 1.0) were stride time and length, step length symmetry, and *RMS* of mode 2 and 3 (reflecting the amplitude drop in the paretic and non-paretic leg). Regression coefficients for changes in stride time, stride length and step length symmetry were negative, indicating that an increase in these parameters was associated with a decrease in energy cost. The opposite was the case for *RMS* of mode 2 and 3, which showed a positive regression coefficient, indicating that decreases in *RMS* were associated with a decrease in energy cost.

**Table 4:** Regression coefficients and VIP scores of the PLS regression analysis

	Regression coefficient	VIP score
Spatiotemporal step parameters		
Stride time	−0.19	2.19*
Stride length	−0.18	1.93*
Step length asymmetry	−0.17	1.74*
Step width	−0.06	0.22
Step width variability	0.03	0.07
Muscle activation parameters		
RMS 1	−0.03	0.01
RMS 2	0.18	1.99*
RMS 3	0.20	2.51*
Variance of relative phase	0.09	0.51
CAI GM_TA paretic	−0.09	0.47
CAI GM_TA nonpar	0.05	0.14
CAI TA_PL nonpar	0.05	0.18

Model was built on difference scores (HOLD - NORM). Variables with a VIP score >1.0, indicating importance for the model, are marked with an asterisk. A negative regression coefficient indicates that an increase in the parameter is associated with a decrease in energy cost and vice versa

## Discussion

In the current study we 1) compared the effects of light touch contact with a handrail and handrail hold on the energy cost, step parameters, and muscle activity during treadmill walking in stroke survivors, and 2) examined which changes in step parameters and/or muscle activity were associated with the potential difference in energy cost. The results provided a clear answer regarding the first research question. Use of a handrail yields a reduction in energy cost, and major changes in both step parameters and muscle activity, but not when the handrail was only touched lightly. The latter only caused minor changes in step width. It thus appears that mechanical support, and not somatosensory feedback as provided with light touch contact, is responsible for the beneficial effects of handrail hold on the energy cost of walking in stroke survivors.

There are several possible reasons why light touch contact did not have the expected facilitating effect on balance control. The original idea that it could came from studies showing a decrease in center-of-mass movement with light touch during upright standing [9, 10]. However, Riley et al. [39] suggested that light touch does not facilitate balance control, but may instead be regarded as a ‘suprapostural’ task that requires precise movement of the center-of-mass to comply with the task instructions regarding the allowed force. From this point of view, the center-of-mass movement serves the precision task, instead of the other way around. This precision aspect may even be amplified by the fact that participants were instructed to use the rail in a continuous fashion instead of intermittently when needed. Two previous studies examining the effects of light touch using a cane in stroke survivors concluded that light touch contact had similar stabilizing effects as force contact during walking in stroke survivors, based on the reductions in pelvic acceleration which were the same for the two ways of contact [12, 26]. However, other gait parameters did not show a change with cane use at all (neither with force contact nor light touch contact), and reductions in EMG amplitude were larger for force contact than for light touch contact. All in all, the facilitating effect of light touch contact with respect to balance control in stroke patients might be contested.

Unlike light touch, handrail hold resulted in a reduction in energy cost (11.9 %), which was slightly lower than in our previous study with stroke survivors (on average 16 %) [7]. Handrail hold also resulted in major changes in step parameters and quantitative and qualitative changes in muscle activity: a significantly increased stride time and length, improved step length symmetry, decreased step width and step width variability, a decrease in the overall muscle activity as evidenced by lower *RMS* for all three relevant modes, a tendency towards a more constant timing of muscle activation, as evidenced by the decreased relative phase variance, and decreased co-activation. Based on the regression analysis the changes in stride time, stride length, step length symmetry and the decrease in muscle activity for mode two and three were most strongly associated with the reduction in energy cost.

The changes in step parameters are consistent with a more efficient use of the pendulum-like characteristics of the legs. Able-bodied people tend to walk at a step frequency-length combination that minimizes the metabolic cost of walking, close to the predicted resonant frequency of the legs, which has been suggested to require minimal muscular activation [40]. Likewise, preferred step width in able-bodied people is similar to the energetically optimal step width. In contrast, stroke survivors often walk with shorter stride lengths and times, and larger step-widths than able-bodied people [41], which may be viewed as a direct consequence of impaired neuromotor control, but also as a strategy to increase the margins of stability during walking [42, 43]. Providing balance support in the form of a handrail artificially enhances balance control and increases the base of support, which might allow stroke survivors to walk at a more optimal step width and step frequency-length combination, requiring less muscle activation. Results from the regression analysis indicated that the sagittal plane gait changes contributed more to the reduction in energy cost than the frontal plane gait changes. The larger influence of stride length (and time) is not surprising, since it has previously been shown that metabolic rate increases with the square of step width, but with the fourth power of step length [18, 17]. Therefore, stride length changes may have overshadowed the influence of step width.

The present results further indicated that the use of a handrail did not induce a major reorganization of neuromuscular coordination. By investigating the underlying patterns of activation using PCA, rather than changes in individual muscle activities, both quantitative and qualitative changes in neuromuscular control could be assessed. Some qualitative changes were observed in terms of improved constancy and decreased co-activation, which may well reflect a more efficient activation pattern. But the factor most strongly related to the change in metabolic energy expenditure was a decrease in *RMS* for all three relevant modes, as opposed to a reweighting of the modes, indicated that handrail hold mainly resulted in a quantitative change in muscle activation (in the form of an overall amplitude drop), without a reorganization of the modes. Presumably, major qualitative changes in neuromuscular control, which require a certain degree of motor learning, are more likely to occur on longer time-scales, particularly in stroke survivors in whom motor control and learning may be affected by cortical damage and reorganization [44]. But, even in the long run, functional changes in gait kinematics of stroke patients may occur with persisting abnormal muscle activation patterns [45, 46]. The present results complement these previous findings by showing that qualitative changes in neuromuscular activation patterns are not necessary to induce functional improvement in gait kinematics and gait economy.

It should be noted that the effect of using a handrail might not solely originate from facilitation of balance control. The handrail may be used to generate propulsive forces in the fore-aft direction, which could be instrumental in increasing stride length and time, and improving step length symmetry. Likewise, using the handrail for (partial) body weight support may allow subjects to spend more time in single limb support, which could also result in an increased stride time and length. However, data on handrail forces presented in the Additional files 1 and 2 shows that the exerted forces during HOLD were rather low in all directions. Forces were largest in the vertical direction, but even in this direction the 95^th^ percentile of the force over the trial was on average only about 6.7 % of the body weight of the participant. Hence, we deem it unlikely that enhanced propulsion or body weight support played a prominent role in the effects on the energy cost of walking or the observed gait changes.

The present study has several limitations that may restrict the generalizability of the results to walking in daily life. First of all, the study was carried out on a treadmill, which, although biomechanically equivalent, is not identical to walking over ground [47]. Also, handrail hold is not the same as using a cane or other walking aid in daily life. For instance, mechanical work is required for holding and carrying a cane, but not for holding a handrail. Therefore these changes in energy cost with cane use are likely to be smaller than with handrail hold. Related to this, holding or touching a handrail constrains movement of the participant over the belt, which could limit step variability. However, since no effects on step variability were present in the light touch condition this does not appear to have influenced the present study. Lastly, due to the strict inclusion criteria (e.g., receiving therapy for stroke-related gait impairments, and able to walk 5 minutes on a treadmill), only a narrow band of the stroke survivors were eligible for this study, limiting in principle the generalizability of results.

These limitations notwithstanding, the study has important clinical implications. First, the reduction in energy cost per meter walked by means of balance support, via a cane or a handrail may allow patients to walk further, increasing available practice time during rehabilitation or increasing walking distance in daily life. Moreover, even though therapists sometimes have reservations prescribing a cane for fear of detrimental effects on the gait pattern [48], using the handrail had beneficial effects on the gait pattern. Thus a handrail, or a cane, may be considered an important instrument, both in therapy and in daily life to improve the gait pattern of stroke survivors. Extending these results further, balance training appears to deserve a prominent role in rehabilitation not only for reducing fall risk but also for improving gait economy. Investigating the effects of balance training on the gait pattern and gait economy represents an interesting direction for future research. Second, the lack of effect of light touch implies that providing only somatosensory information does not improve the gait pattern of stroke patients. In rehabilitation, therapists often provide light touch cues through manual facilitation at the pelvis or the paretic leg to improve the gait pattern. This passive touch, often provided at a specific instant in the gait cycle and with a specific direction, is however very different from the continuous active touch provided in our experiment. Therefore, the lack of effect of the light touch condition in the present study by no means implies that such facilitation methods are ineffective in improving the gait pattern.

## Conclusion

The energy cost of walking in stroke survivors is effectively reduced by means of handrail support, but not when only light touch of the handrail is allowed. Handrail hold resulted in a normalization of step parameters and decreased muscle activity without major qualitative changes in muscle coordination. We speculate that the biomechanical advantage of using a handrail, and possibly other handheld assistive devices for balance control (i.e. the larger base of support, and the potential of using the arm for balance corrections) may allow stroke survivors to adopt a more optimal step length (and width), which requires less muscle activity and, hence, comes with an energetic advantage.

## Additional files

Additional file 1: Figure S1. Forces exerted on the handrail during the stride cycle during touch (upper graphs) and hold (lower graphs) condition. Stride cycle starts and ends with initial contact of the nonparetic leg. Black line represents the group mean of the ensemble averaged force. Shaded area represents the corresponding standard deviation. Force data were filtered with a 4^th^ order Savitzky-Golay filter with frame size 41. Additional file 2: Table S1. 95^th^ percentile of absolute forces (N) exerted on the handrail during TOUCH and HOLD. Table shows additional information regarding the forces exerted on the handrail during the experimental conditions.
