# Supplementary material for: Effects of handrail hold and light touch on energetics, step parameters, and neuromuscular activity during walking after stroke
Source: J Neuroeng Rehabil. 2015 Aug 23;12:70. doi: 10.1186/s12984-015-0051-3 (PMC4546819; doi:10.1186/s12984-015-0051-3)
Supplement: Additional file 2: Table S1. — 95th percentile of absolute forces (N) exerted on the handrail during TOUCH and HOLD. Table shows additional information regarding the forces exerted on the handrail during the experimental conditions. [file 12984_2015_51_MOESM2_ESM.docx]

**Additional Table 1: 95^th^ percentile of absolute forces (N) exerted on the handrail during TOUCH and HOLD**

|  | **TOUCH** | **HOLD** |
| --- | --- | --- |
| Vertical | 4.74 (1.99) | 95.63 (58.84) |
| Antero-posterior | 2.00 (0.63) | 14.00 (5.42) |
| Mediolateral | 5.41 (0.82) | 10.89 (7.51) |
| Resultant | 6.79 (1.76) | 97.42 (58.24) |

Data were Savitzky-Golay filter (4^th^ order, frame size 41), to reduce input noise. During normal and light touch trials some subjects momentarily increased the amount of force on the handrail when they had to change their fore-aft position on the treadmill (in order to stay on the treadmill). To diminish the effect of these instantaneous peak forces we used the 95^th^ percentile of the force as a cut-off value.
